# Heritability of Subcortical Grey Matter Structures

**DOI:** 10.3390/medicina58111687

**Published:** 2022-11-21

**Authors:** David Strelnikov, Amirreza Alijanpourotaghsara, Marton Piroska, Laszlo Szalontai, Bianka Forgo, Zsofia Jokkel, Alíz Persely, Anita Hernyes, Lajos Rudolf Kozak, Adam Szabo, Pal Maurovich-Horvat, David Laszlo Tarnoki, Adam Domonkos Tarnoki

**Affiliations:** 1 Medical Imaging Centre, Semmelweis University, 1082 Budapest, Hungary; 2 Department of Radiology, Faculty of Medicine and Health, Örebro University, 702 81 Örebro, Sweden

**Keywords:** subcortical, volumetry, twins, heritability, MRI, CAT12, volBrain

## Abstract

*Background and Objectives*: Subcortical grey matter structures play essential roles in cognitive, affective, social, and motoric functions in humans. Their volume changes with age, and decreased volumes have been linked with many neuropsychiatric disorders. The aim of our study was to examine the heritability of six subcortical brain volumes (the amygdala, caudate nucleus, pallidum, putamen, thalamus, and nucleus accumbens) and four general brain volumes (the total intra-cranial volume and the grey matter, white matter, and cerebrospinal fluid (CSF) volume) in twins. *Materials and Methods*: A total of 118 healthy adult twins from the Hungarian Twin Registry (86 monozygotic and 32 dizygotic; median age 50 ± 27 years) underwent brain magnetic resonance imaging. Two automated volumetry pipelines, Computational Anatomy Toolbox 12 (CAT12) and volBrain, were used to calculate the subcortical and general brain volumes from three-dimensional T1-weighted images. Age- and sex-adjusted monozygotic and dizygotic intra-pair correlations were calculated, and the univariate ACE model was applied. Pearson’s correlation test was used to compare the results obtained by the two pipelines. *Results*: The age- and sex-adjusted heritability estimates, using CAT12 for the amygdala, caudate nucleus, pallidum, putamen, and nucleus accumbens, were between 0.75 and 0.95. The thalamus volume was more strongly influenced by common environmental factors (C = 0.45−0.73). The heritability estimates, using volBrain, were between 0.69 and 0.92 for the nucleus accumbens, pallidum, putamen, right amygdala, and caudate nucleus. The left amygdala and thalamus were more strongly influenced by common environmental factors (C = 0.72−0.85). A strong correlation between CAT12 and volBrain (r = 0.74−0.94) was obtained for all volumes. *Conclusions*: The majority of examined subcortical volumes appeared to be strongly heritable. The thalamus was more strongly influenced by common environmental factors when investigated with both segmentation methods. Our results underline the importance of identifying the relevant genes responsible for variations in the subcortical structure volume and associated diseases.

## 1. Introduction

The amygdala, nucleus accumbens, pallidum, putamen, caudate nucleus, and thalamus represent prominent members in countless functional networks and play important roles in cognitive, affective, social, and motoric functions in humans [1,2]. Changes in the volume of these subcortical structures have been associated with age, sex, ethnicity, and various lifestyle factors such as smoking and exercise habits [3,4,5,6,7,8,9,10]. Furthermore, they have also been linked with a multitude of different disorders. Alzheimer’s disease has been shown to exhibit a decrease in the subcortical structure volume. Moreover, the decrease in volume appeared to be correlated with the severity of the cognitive impairment exhibited by patients [11]. Parkinson’s disease showed an association with a decrease in the volume of the amygdala and dorsal striatum (caudate and putamen) whilst sparing the ventral striatum [12]. Other disorders associated with a significant change in the subcortical structure volume include schizophrenia [1], bipolar disorder [13], attention deficit hyperactivity disorder [14], major depressive disorder [15], and Huntington’s disease [16]. The variation in the volume of these structures in the disease states further emphasizes their significance and the importance of understanding the factors that influence their development.

It has been consistently shown that general brain volumes such as the total intra-cranial volume (TIV), total brain volume (TBV), and total grey matter (GM) and white matter (WM) volumes are strongly heritable [17,18,19,20]. Even though a moderate-to-high genetic contribution has also been seen for subcortical structures, the results have demonstrated weaker heritability estimates and a wider variation compared with general brain volumes [17,21,22]. A large sample examining several European and American cohorts found that the amygdala and thalamus had the weakest genetic influence among the subcortical structures [17,23]. However, in a study by Christova et al., the heritability of the left and right thalamus was 91% and 99%, respectively. They also calculated a heritability of 94% for the right amygdala [19]. Similarly, a study examining twins from the Netherlands showed a strong genetic association for the thalamus [24]. Blokland et al. described that larger brain volumes were associated with higher heritability estimates. This was suggested to be a result of a greater measurement error (a bias in regional partitioning) for the smaller subcortical structures [17].

The heritability of brain volumes also varies with age. A review by Batouli et al. found that heritability estimates for general brain volumes increased from birth to early adulthood. This was followed by a subsequent decline with an increasing age. Yet, the heritability of subcortical brain structures has shown differing results. A few have shown a steady decrease in heritability with age [25]. Others, for example, have shown that the amygdala appears to have a steady increase in heritability with age. However, this review was based mainly on cross-sectional studies, which have limitations. In addition, the number of studies examining subcortical structures and participants were much lower than for general brain volumes [26,27].

Even though many studies have investigated the heritability of general brain volumes (i.e., TIV, TBV, total GM, and WM volumes), subcortical structures have received much less attention and the results of heritability analyses are inconsistent throughout the different studies. Even though it is generally agreed that they are heritable, the strength of the genetic association varies widely between different studies. The aim of our study was to examine the heritability of the subcortical structures in a healthy Caucasian twin population. Twin studies provide the ideal study model to assess the contribution of genetic versus environmental factors to a certain phenotype. Furthermore, they allow us to distinguish between common and unique environmental contributions, which cannot be achieved using family studies.

## 2. Materials and Methods

### 2.1. Study Participants

This study examined 118 healthy adult asymptomatic Caucasian twins (56 pairs and 2 triplets) from the Hungarian Twin Registry [28] with no history of previous cerebrovascular or neurodegenerative diseases. The triplets were regarded as three different twin pairs for statistical purposes. This resulted in a total of 62 twin pairs, of which 43 were monozygotic (MZ) and 19 were dizygotic (DZ). Two twin pairs were excluded from the study because of missed appointments, one pair was excluded due to an inadequate imaging quality, and one pair was excluded due to an exclusion of opposite-sex DZ twin pairs. The median age of all participants was 50 ± 27 years, and the proportion of female to male participants was 71.2% to 28.8%, respectively. The study was approved by the local Ethical Committee (Semmelweis University, TUKEB 189-1/2014, amendments on 10 October 2016 and 7 December 2018). All participants signed an informed consent form. The tenets of the Declaration of Helsinki were followed. The zygosity classification was determined using a seven-part self-reported questionnaire [29]. Information about history and risk factors was obtained using a questionnaire and included height, body weight, body mass index (BMI), smoking, hypertension, hyperlipidemia, and diabetes. Former smokers were also included in the smoking category.

The participants underwent brain magnetic resonance imaging (MRI) examinations if no contraindications were present. The exclusion criteria included an immunosuppressive or immunomodulant therapy in the past month, chemotherapy in the past year, major surgery in the past two months, transfusion of blood or blood products in the past two months, current pregnancy, or breastfeeding. Likewise, participants with pacemakers, implantable cardioverter defibrillators or other implanted devices, magnetic metal foreign bodies, and claustrophobia were excluded. The examinations were performed at the Semmelweis University Medical Imaging Centre, Department of Neuroradiology, in Budapest, Hungary.

### 2.2. MRI Acquisition

Three-dimensional (3D) T1-weighted images were obtained for all participants. No contrast agent was administered. All studies were performed at Semmelweis University MR Research Centre using a Philips Ingenia 3T scanner (Philips Healthcare, Best, The Netherlands). The following imaging parameters were used in the Philips scanner: 140/9000 ms TE/TR, 88° flip angle, 290 × 336 × 336 matrix, 0.8333 × 0.8333 in-plane resolution, and 0.6 mm slice thickness.

### 2.3. Image Processing

The absolute volumes of the subcortical structures, including the nucleus accumbens, amygdala, caudate nucleus, pallidum, putamen, and thalamus, were measured using two different software pipelines. Similarly, the measurement of global volumes of GM, WM, and TIV was also carried out.

A DCM2NII converter was used (http://www.mricro.com, mricron; Chris Rorden, Columbia, SC, USA, accessed on 19 September 2021) in order to convert the 3D T1-weighted images from DICOM (Digital Imaging and Communications in Medicine) format to NIfTI (Neuroimaging Informatics Technology Initiative; http://nifti.nimh.nih.gov/) format. All subsequent image processing was carried out in this image format [30].

#### 2.3.1. Volume Measurement Using Computational Anatomy Toolbox (CAT12) for Statistical Parametric Mapping (SPM12)

**Figure 1:**
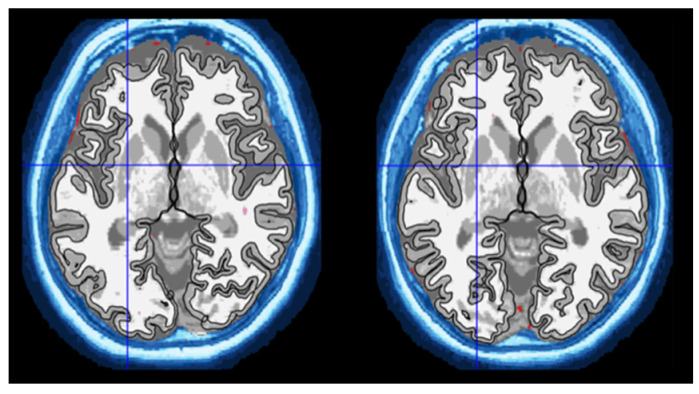
The 3D T1-weighted magnetic resonance images of 69-year-old monozygotic twins taken at the Medical Imaging Centre, Semmelweis University. The images were segmented using Computational Anatomy Toolbox 12. The subcortical structures were delineated and their volumes were calculated.

The CAT12 toolbox (http://www.neuro.uni-jena.de/cat, accessed on 8 October 2021) was used to segment the images and calculate the relevant volumes [31]. CAT12 is an extension pipeline of the classical SPM12 [32] software. However, it uses a completely different segmentation approach. The 3D T1-weighted images were pre-processed and then segmented in an automated manner using the AMAP (Adaptive Maximum A Posterior) technique, which did not require the need for prior information on the tissue probabilities. The segmentation process classified the images into separate GM, WM, and cerebrospinal fluid (CSF) components. This was followed by the use of template probabilistic brain atlases to calculate the region of interest (ROI) volumes in cubic centimeters (cm^3^) for the specific brain structures. The Neuromorphometrics atlas (Neuromorphometrics Inc., Somerville, MA, USA) was used to calculate the volumes of the subcortical brain structures [33,34,35]. An example of the segmentation process using CAT12 can be seen in Figure 1.

#### 2.3.2. Volumetric Measurement Using volBrain

**Figure 2:**
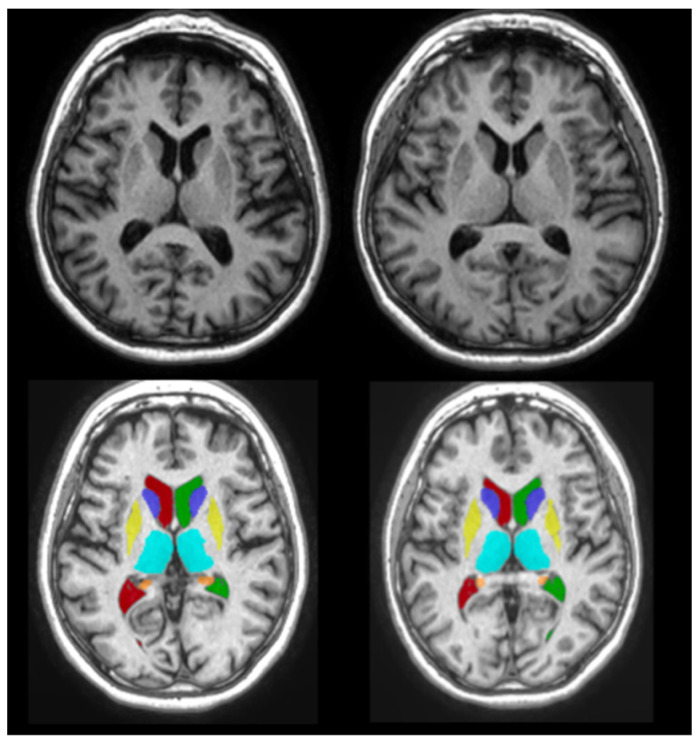
The 3D T1-weighted magnetic resonance images of 69-year-old monozygotic twins taken at the Medical Imaging Centre, Semmelweis University. The images were segmented using volBrain. The subcortical structures were delineated and their volumes were calculated.

**Figure 3:**
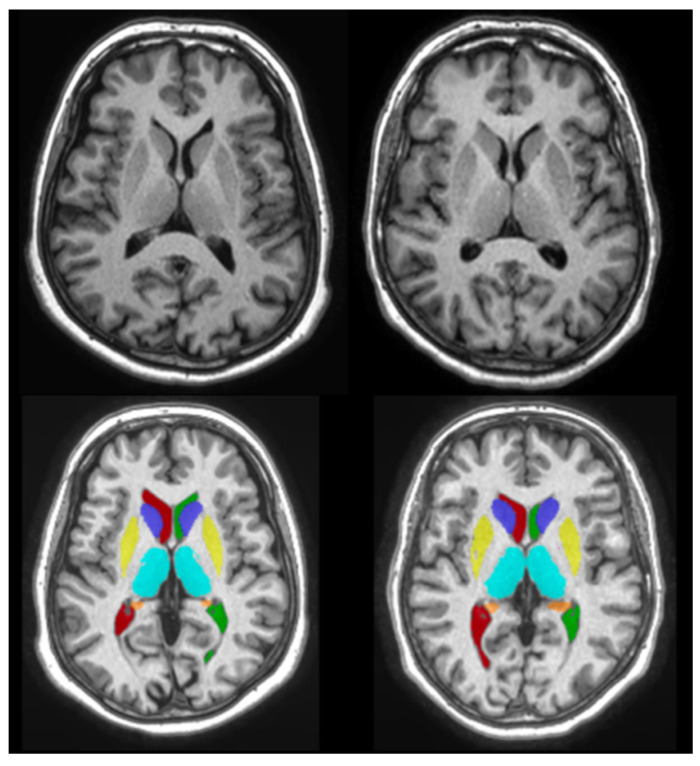
The 3D T1-weighted magnetic resonance images of 68-year-old dizygotic twins taken at the Medical Imaging Centre, Semmelweis University. The images were segmented using volBrain. The subcortical structures were delineated and their volumes were calculated.

The volBrain pipeline (https://www.volbrain.upv.es, accessed on 20 November 2020) was used to calculate the volume of the subcortical brain structures. volBrain is an automated MRI brain volumetry system that requires no human interaction to perform all tasks and generate a report [36,37]. It started by de-noising the scans, then performed an inhomogeneity correction, a Montreal Neurologic Institute (MNI) space registration, an intensity normalization, and an intra-cranial cavity extraction. Tissue segmentation was then performed using a multi-template fusion atlas method based on a library built from the manual segmentation of 50 subjects [38]. The volume was then measured for the relevant structures in cubic centimeters (cm^3^) taking into account the age and sex for the expected values of normal development. An example of the segmentation process using volBrain can be seen in Figure 2 and Figure 3. Additional information about the segmentation pipelines is provided in Supplement S1.

### 2.4. Statistical Analysis

#### 2.4.1. Descriptive Statistics

The continuous variables were examined for normal distributions using the Shapiro–Wilk test. If they were found to be normally distributed, the means of the variables were compared between the MZ and DZ twins using the independent samples t-test. These variables included the BMI and were expressed as the mean ± standard deviation (SD). For the non-normally distributed variables, a comparison was made using the non-parametric Mann–Whitney U-test. This was applied for the age of participants, which was expressed as the median ± interquartile range (IQR). The categorical variables such as sex, diabetes, smoking, hypertension, and hyperlipidemia were compared using the chi-squared test and expressed as frequencies and percentages. Furthermore, the absolute volumes of the subcortical structures were compared between the MZ and DZ twins. A *p*-value less than 0.05 was considered to be significant. All descriptive statistical analyses were conducted using Statistical Package for the Social Sciences (SPSS) software (International Business Machines Corporation, IBM Corp., IBM SPSS Statistics, Armonk, NY, USA).

#### 2.4.2. Heritability Analysis

A phenotypic variance between individuals is attributed to the sum of genetic and environmental effects. Genetic influences are further subdivided into additive genetic factors (A), including polygenetic inheritance, and dominant genetic factors (D), such as epistasis. Likewise, environmental factors can be shared (C) and unique (E). Common environmental factors refer to the shared familial environment experienced by twins, which includes a common childhood diet, exposure to parental smoking, air pollution, and even sharing a womb. Non-shared environmental factors refer to unique exposures and experiences for individual twins but not their siblings; for example, smoking habits, physical activity, occupational exposures, and different illnesses [39].

Classical twin studies rely on the idea that MZ twins share roughly 100% of their genetic information whereas DZ twins share only 50% on average. Furthermore, both MZ and DZ twins share all their common environmental factors and none of their unique ones. Heritability estimates were determined by comparing the intra-pair correlations in the MZ and DZ twins. If the intra-pair correlations were higher in the MZ than the DZ twins, this indicated a genetic influence. However, if the intra-pair correlations were similar in both the MZ and DZ twins, the variance was attributed to environmental factors [40]. Univariate quantitative genetic modeling was used to split the variance into additive genetic (A), common environmental (C), and unique environmental influences (E). The results were adjusted for age and sex. Based on this model, dominant genetic factors (D) and common environmental factors (C) could not be simultaneously calculated because of the confounding effects. Hence, the best fitting model (ACE) was used. A reduced AE or CE model was considered for the variables with negligible genetic or common environmental influences, respectively. A likelihood-ratio test was performed to compare the models and *p*-values higher than 0.05 indicated that we could not prove a significant difference between the base and reduced models, justifying the use of the more parsimonious AE or CE model.

#### 2.4.3. Correlation of Subcortical Structure Volume Measured Using Different Software

The obtained volumetric results were tested for a normal distribution. A correlation analysis was performed using Pearson’s correlation test to compare the two volumetry methods. The correlation coefficients (*r*) and *p*-values were reported. *p*-Values less than 0.05 were considered to be significant for an existing correlation between the two methods. The general brain volumes were omitted in this analysis to emphasize the subcortical structures as these smaller volumes were more challenging to the segment reliably.

## 3. Results

### 3.1. Descriptive Statistics

The median age was 46 and 64 years in the MZ and DZ groups, respectively. There was no significant difference between the two groups for BMI, smoking, diabetes, hypertension, and hyperlipidemia. However, a significant difference was observed for age (*p* = 0.03) between the two groups. Due to the mean age difference in the MZ and DZ twins, we compared models where age was separately regressed out on the MZ and DZ twins. This showed no significant difference when compared with the base models, where a regression on age and sex was simultaneously carried out in the MZ and DZ groups. Furthermore, the examined subcortical structure volumes did not show a significant difference between the MZ and DZ twins. Table 1 shows the characteristics of the MZ and DZ twin study populations.

### 3.2. Results for Volume Measurements Using CAT12

#### 3.2.1. Intra-Pair Correlation Coefficients for Subcortical and General Brain Volumes Using CAT12

The age- and sex-adjusted intra-pair correlation coefficients in the MZ twins were higher than in the DZ twins for all the subcortical brain volumes and most of the general brain volumes measured using CAT12. A higher DZ intra-pair correlation was seen only for the total CSF volume (rMZ = 0.67 and rDZ = 0.73). The MZ intra-pair correlation was much higher than the DZ intra-pair correlation for the total GM, bilateral nucleus accumbens, bilateral amygdala, left pallidum, and bilateral putamen. Table 2 shows the results of the intra-pair correlation analysis of the subcortical and general brain volumes in the MZ and DZ twins.

#### 3.2.2. Univariate Model Analysis for Subcortical and General Brain Volumes Using CAT12

The age- and sex-adjusted univariate analysis demonstrated a strong heritability (A) for all general and subcortical brain volumes, excluding the right and left thalamic volumes. The more parsimonious AE model was applied for these structures due to negligible common environmental (C) contributions; the model fit demonstrated no significant difference in comparison with the more general ACE model. For the right thalamic volume, a CE model was applied and demonstrated a strong common environmental (C) contribution (0.73) and a moderate unique environmental (E) contribution (0.27). For the left thalamic volume, the ACE model was the best fit, with moderate genetic (0.40) and common environmental (0.45) contributions. A unique environmental variance was a minor contributor for all variables. Table 3 shows the results of the univariate model heritability analysis for the subcortical and general brain volumes using CAT12 in twins. Additional information is provided in Table S1.

### 3.3. Results for Volume Measurements Using volBrain

#### 3.3.1. Intra-Pair Correlation Coefficients for Subcortical and General Brain Volumes Using volBrain

The intra-pair correlation coefficients in the MZ twins were higher than in the DZ twins for all the general and most of the subcortical brain volumes. A higher DZ intra-pair correlation was only seen for the right thalamic volume (rMZ = 0.68 and rDZ = 0.83). Furthermore, an equal MZ and DZ intra-pair correlation was seen for the left thalamus. The MZ intra-pair correlation was much higher than the DZ intra-pair correlation for the TIV, total GM, bilateral nucleus accumbens, and bilateral putamen. Table 4 shows the results of the intra-pair correlation analysis of the subcortical and general brain volumes using volBrain in the MZ and DZ twins.

#### 3.3.2. Univariate Model Analysis for Subcortical and General Brain Volumes Using volBrain

The age- and sex-adjusted univariate analysis demonstrated a strong heritability (A) for all general and the majority of subcortical brain volumes, excluding the left amygdala and bilateral thalamic volumes. The more parsimonious AE model was applied for these structures due to negligible common environmental (C) contributions; the model fit demonstrated no significant difference in comparison with the more general ACE model. For the left amygdala and bilateral thalamus, the CE model was applied and demonstrated a strong common environmental (C) contribution (0.76, 0.85, and 0.72 for the left amygdala, left thalamus, and right thalamus, respectively). A unique environmental variance was a minor contributor for all variables. Table 5 shows the results of the univariate model heritability analysis for the subcortical and general brain volumes using volBrain in the twins. Additional information is provided in Table S2.

### 3.4. Correlation of Subcortical Structure Volume Measured Using Different Software

The results of the Pearson’s correlation test for the measurement of the subcortical volumes using CAT12 and volBrain software are shown in Table 6. The correlation coefficient (*r*) was greater than 0.5 for all variables, indicating a strong correlation. The left caudate nucleus, bilateral pallidum, and putamen all had values above 0.9, indicating a very strong correlation. The correlation coefficients were lowest (< 0.8) for the left and right thalamus. The *p*-values were significant for all variables, indicating a significant existing association between the methods.

## 4. Discussion

In our study, we aimed to examine the heritability of six subcortical brain volumes (the amygdala, caudate nucleus, pallidum, putamen, thalamus, and nucleus accumbens) and four general brain volumes (TIV, total GM, WM, and CSF volume) in a population of healthy adult Hungarian twins. We chose to concentrate on the subcortical structures because the results for these appeared to be much more variable in previous studies than the results for the heritability of bigger, more general brain volumes. We used two automated systems to calculate the volumes—namely, CAT12 and volBrain—and performed a correlation analysis to compare the results produced by the two software programs.

The results of the heritability analysis using CAT12 demonstrated strong genetic contributions for all examined volumes, except for the left and right thalamic volumes. These results were in the range of 0.75–0.95. The results for the general brain volumes were similar, if slightly higher than in previously examined studies; however, the heritability of the subcortical structures appeared to be much stronger than that shown in previous studies, which ranged between 0.34 and 0.99 [17,18,23,41,42]. The variance in the left thalamic volume appeared to be equally governed by genetic and common environmental factors whereas the variance in the right thalamic volume appeared to rely mostly on common environmental contributions. Similarly, lower heritability estimates for thalamic volumes have been found in several studies [17,21,23,43]. However, others have found that the thalamus was a highly heritable, if not the most heritable, subcortical structure [19,44,45]. The generally lower heritability of the subcortical structures seen in previous studies may be explained by the smaller size and higher complexity of these structures. This makes it harder to accurately segment them and increases the error in their measurement [17]. However, the strong common environmental contribution to the thalamic volumes in our population suggested a shared influence in utero or during early life that affected both twins. This may have impact the thalamic development and, in turn, could be predisposed to an early age-related decline in cognition and memory [46]. Further studies should attempt to replicate these results in a larger population, and possible shared environmental factors should be identified.

Similar to the results from CAT12, a strong heritability was seen using volBrain for all general brain volumes and most subcortical structures. The left and right thalamic volumes appeared to be mainly influenced by common environmental factors. Likewise, the left amygdala showed a weak heritability and a strong influence by common environmental factors. Similar results for the amygdala have been seen in several studies [43,44,47].

When examining the intra-pair correlations between the MZ and DZ twins with both software packages, several structures demonstrated an MZ intra-pair correlation that was more than twice the DZ intra-pair correlation. This suggested that the genetic contribution to the variance was not exclusively additive, but included a dominant genetic influence. In 2015, Hibar et al. performed a genome-wide association study and discovered seven single nucleotide polymorphisms (SNPs) associated with the putamen, caudate, and hippocampal volumes [48]. More recently, Satizabal et al. examined the genetic variants associated with seven subcortical structures (the nucleus accumbens, amygdala, caudate nucleus, putamen, globus pallidus, thalamus, and brain-stem) in 37,741 individuals. They discovered 48 significant SNPs, of which 26 were located within genes and 22 were located in intergenic regions. Additionally, they found that 26 candidate genes could influence more than one structure. For example, significant SNPs near *KTN1* were also associated with the volume of the nucleus accumbens, caudate nucleus, and globus pallidus, suggesting that this genomic region may have an important role in determining multiple subcortical brain volumes during development [23,49]. Subcortical structures are known to be associated with a multitude of neuropathological conditions. A volumetric analysis is already being used in clinical practice for disorders such as Alzheimer’s disease and other forms of dementia. By demonstrating that these structures are highly heritable, we invite further studies to explore the specific genes that contribute to their development. This could allow the possibility of future genetic screening for subcortical structure dysfunctions and disease [50,51].

Our results demonstrated a strong correlation between the volumes calculated using CAT12 and volBrain, with a significance of *p* < 0.001 for all values. This was in line with previous results, which compared these pipelines with more well-known software packages available. The weakest correlation was seen for the right and left thalamic volumes. This could be the result of a difference in the segmentation of these structures by the automated software [47,52]. These results may justify the use of simpler, online, and fully automated pipelines such as volBrain for further research into subcortical volumes.

Our limitations included a relatively small sample size of twins. Even though we achieved results with a sufficient level of significance (*p* < 0.05), it would be important to perform this study with a larger twin population. We also found a significant difference in the average age between the MZ and DZ groups. The volume of subcortical structures changes with age. Therefore, a significant difference in age between the MZ and DZ groups could have skewed our results, despite our attempt to correct for age during the statistical analysis. As CAT12 and volBrain work in an automated manner, our segmentation could not be manually corrected. This has the advantage of similar measurements without observer bias, but it also has the disadvantage of not being able to distinguish potential artifacts. We attempted to overcome this limitation by manually reviewing the images for visible artifacts prior to initiating the segmentation. Automated platforms have been shown to have difficulty in segmenting small, more complex structures, which could have led to errors in the measurement of volume. We attempted to correct for that by using two different systems of measurement. Lastly, in our study, we did not correct the subcortical structure volume for TIV. This is frequently performed to correct for variations in head sizes between individuals. As we were measuring structures in twins, we did not expect the changes in TIV to significantly impact our heritability results.

## 5. Conclusions

Our results showed a strong heritability for all general brain volumes (TIV, total GM, WM, and CSF volume) and most of the subcortical structures examined (the amygdala, caudate nucleus, pallidum, putamen, and nucleus accumbens) in healthy adult twins on a 3T MRI scanner. The thalamus appeared to be the least heritable structure in both methods of measurement, and appeared to be strongly influenced by common environmental factors. Additionally, the left amygdala appeared to be less heritable, but only for the volBrain results. We used two different software pipelines to segment and measure our volumes, and found a significant correlation between their results. Using the ACE/AE/CE model, we estimated a strong genetic (additive and possibly dominant) contribution to the development of these volumes. Our results should stimulate further research into specific genes that could be responsible for the development and maintenance of subcortical volumes with age and disease. This could present future opportunities for the early screening of disorders affecting the subcortex. Furthermore, attempts should be made to identify the shared environmental factors that influence the development of thalamic volumes in healthy adult twins. These results should be further verified in a larger twin population.

## Figures and Tables

**Table 1:** Characteristics of the MZ and DZ twin study populations. BMI: body mass index; DZ: dizygotic; MZ: monozygotic. Data are shown as mean ± standard deviation with continuous variables that had a normal distribution (BMI), and as median ± interquartile range with continuous variables that had a non-normal distribution (age). ^†^: Results of the non-parametric Mann–Whitney U-test. All other *p*-values for continuous variables were calculated using the independent samples t-test. *p*-Values for dichotomous variables were calculated using the chi-squared test. *p*-Values less than 0.05 were considered to be significant. Significant results are marked with an asterisk *.

	Total (*n* = 118)	MZ (*n* = 86)	DZ (*n* = 32)	*p*-Value
Zygosity (*n*MZ:*n*DZ)	86:32	-	-	-
Sex (male:female)	32:86	24:62	10:22	0.90
Age (years)	50 ± 27	46 ± 23	63.5 ± 29.5	0.04 ^†,^*
Body mass index (kg/m^2^)	24.4 ± 4.3	24.3 ± 4.6	24.5 ± 3.4	0.86
Smoking, *n* (%)	12 (14.3)	9 (15.3)	3 (12.0)	0.70
Diabetes, *n* (%)	6 (7.1)	4 (6.8)	2 (8.0)	0.84
Hypertension, *n* (%)	23 (27.4)	15 (25.4)	8 (32.0)	0.54
Hyperlipidemia, *n* (%)	20 (23.8)	16 (27.1)	4 (16.0)	0.27

**Table 2:** Intra-pair correlation coefficients of subcortical and general brain volumes using CAT12 in MZ and DZ twins. Results and 95% confidence intervals were reported (95% CI). All volumes were measured in cubic centimeters (cm^3^). rMZ: intra-pair correlation coefficient in monozygotic twins; rDZ: intra-pair correlation coefficient in dizygotic twins; TIV: total intra-cranial volume; GM: grey matter; WM: white matter; CSF: cerebrospinal fluid.

	rMZ	rDZ
TIV	0.92 (0.88, 0.95)	0.50 (0.10, 0.72)
Total GM	0.91 (0.85, 0.94)	0.27 (−0.16, 0.59)
Total WM	0.93 (0.90, 0.96)	0.41 (0.01, 0.67)
Total CSF	0.67 (0.46, 0.80)	0.73 (0.49, 0.85)
Right accumbens	0.90 (0.82, 0.94)	0.29 (−0.15, 0.64)
Left accumbens	0.86 (0.76, 0.92)	0.11 (−0.33, 0.52)
Right amygdala	0.87 (0.78, 0.93)	0.36 (−0.08, 0.68)
Left amygdala	0.74 (0.56, 0.85)	0.16 (−0.30, 0.56)
Right caudate	0.87 (0.78, 0.93)	0.66 (0.32, 0.85)
Left caudate	0.90 (0.82, 0.94)	0.44 (0.02, 0.74)
Right pallidum	0.91 (0.85, 0.95)	0.43 (0.02, 0.72)
Left pallidum	0.90 (0.83, 0.95)	0.25 (−0.20, 0.61)
Right putamen	0.90 (0.82, 0.94)	0.20 (−0.24, 0.57)
Left putamen	0.92 (0.86, 0.96)	0.02 (−0.41, 0.44)
Right thalamus	0.74 (0.57, 0.85)	0.73 (0.43, 0.88)
Left thalamus	0.87 (0.78, 0.93)	0.72 (0.42, 0.88)

**Table 3:** Age- and sex-adjusted heritability analysis of general and subcortical brain volumes using CAT12 in twins. Results and 95% confidence intervals were reported (95% CI). *p*-Values less than 0.05 were considered to be significant. A: variance explained by additive genetic factors; C: shared environmental variance component; E: unique environmental variance component; model fit: *p*-value of the chi-squared test based on model log-likelihood comparative test to the base ACE model; TIV: total intra-cranial volume; GM: grey matter; WM: white matter; CSF: cerebrospinal fluid.

	A	C	E	Model Fit (*p*-Value)
TIV	0.93 (0.89, 0.96)	0	0.07 (0.05, 0.11)	1
Total GM	0.93 (0.88, 0.96)	0	0.08 (0.04, 0.12)	1
Total WM	0.93 (0.89, 0.96)	0	0.07 (0.04, 0.11)	1
Total CSF	0.81 (0.67, 0.89)	0	0.19 (0.11, 0.33)	0.92
Right accumbens	0.90 (0.86, 0.94)	0	0.10 (0.06, 0.15)	1
Left accumbens	0.91 (0.83, 0.95)	0	0.09 (0.05, 0.17)	1
Right amygdala	0.86 (0.76, 0.91)	0	0.15 (0.09, 0.24)	1
Left amygdala	0.75 (0.58, 0.86)	0	0.25 (0.14, 0.42)	1
Right caudate	0.87 (0.79, 0.92)	0	0.13 (0.08, 0.21)	0.14
Left caudate	0.91 (0.84, 0.94)	0	0.09 (0.06, 0.16)	1
Right pallidum	0.90 (0.84, 0.94)	0	0.10 (0.06, 0.16)	0.85
Left pallidum	0.93 (0.9, 0.96)	0	0.07 (0.04, 0.13)	1
Right putamen	0.90 (0.82, 0.94)	0	0.10 (0.06, 0.18)	1
Left putamen	0.95 (0.93, 0.97)	0	0.05 (0.03, 0.07)	1
Right thalamus	0	0.73 (0.59, 0.83)	0.27 (0.17, 0.41)	0.74
Left thalamus	0.44 (0.13, 0.91)	0.45 (0, 0.75)	0.11 (0.07, 0.19)	-

**Table 4:** Intra-pair correlation coefficients of subcortical and general brain volumes using volBrain in MZ and DZ twins. Results and 95% confidence intervals were reported (95% CI). All volumes were measured in cubic centimeters (cm^3^). rMZ: intra-pair correlation coefficient in monozygotic twins; rDZ: intra-pair correlation coefficient in dizygotic twins; TIV: total intra-cranial volume; GM: grey matter; WM: white matter.

	rMZ	rDZ
TIV	0.93 (0.89, 0.95)	0.36 (−0.06, 0.64)
Total GM	0.94 (0.89, 0.96)	0.27 (−0.16, 0.58)
Total WM	0.88 (0.81, 0.9)	0.42 (0.02, 0.67)
Total CSF	0.79 (0.64, 0.88)	0.34 (−0.13, 0.71)
Right accumbens	0.72 (0.55, 0.84)	−0.02 (−0.45, 0.42)
Left accumbens	0.79 (0.64, 0.88)	−0.07 (−0.49, 0.38)
Right amygdala	0.83 (0.70, 0.90)	0.59 (0.21, 0.82)
Left amygdala	0.78 (0.64, 0.88)	0.71 (0.40, 0.88)
Right caudate	0.89 (0.80, 0.94)	0.59 (0.21, 0.82)
Left caudate	0.91 (0.84, 0.95)	0.68 (0.34, 0.86)
Right pallidum	0.87 (0.77, 0.92)	0.49 (0.08, 0.76)
Left pallidum	0.85 (0.74, 0.92)	0.50 (0.08, 0.76)
Right putamen	0.92 (0.85, 0.95)	0.17 (−0.28, 0.56)
Left putamen	0.89 (0.81, 0.94)	0.20 (−0.26, 0.58)
Right thalamus	0.68 (0.48, 0.81)	0.83 (0.61, 0.93)
Left thalamus	0.86 (0.76, 0.92)	0.86 (0.69, 0.94)

**Table 5:** Age- and sex-adjusted univariate analysis of general and subcortical brain volumes using volBrain in twins. Results and 95% confidence intervals were reported (95% CI). *p*-Values less than 0.05 were considered to be significant. A: heritability; C: shared environmental variance component; E: unique environmental variance component; model fit: *p*-value of the chi-squared test based on model log-likelihood comparative test to the base ACE model; TIV: total intra-cranial volume; GM: grey matter; WM: white matter.

	A	C	E	Model Fit (*p*-Value)
TIV	0.93 (0.90, 0.96)	0	0.07 (0.04, 0.10)	1
Total GM	0.94 (0.90, 0.96)	0	0.06 (0.04, 0.10)	1
Total WM	0.88 (0.80, 0.92)	0	0.12 (0.08, 0.20)	1
Total CSF	0.74 (0.60, 0.84)	0	0.26 (0.16, 0.40)	0.45
Right accumbens	0.69 (0.49, 0.82)	0	0.31 (0.18, 0.52)	1
Left accumbens	0.78 (0.62, 0.87)	0	0.23 (0.13, 0.38)	1
Right amygdala	0.80 (0.68, 0.88)	0	0.20 (0.12, 0.32)	0.21
Left amygdala	0	0.76 (0.63, 0.85)	0.24 (0.16, 0.37)	0.51
Right caudate	0.88 (0.80, 0.93)	0	0.12 (0.07, 0.20)	0.31
Left caudate	0.91 (0.85, 0.94)	0	0.09 (0.06, 0.16)	0.14
Right pallidum	0.84 (0.75, 0.90)	0	0.16 (0.10, 0.25)	0.37
Left pallidum	0.84 (0.75, 0.91)	0	0.16 (0.10, 0.26)	0.64
Right putamen	0.92 (0.86, 0.95)	0	0.08 (0.05, 0.14)	1
Left putamen	0.90 (0.82, 0.94)	0	0.10 (0.06, 0.18)	1
Right thalamus	0	0.72 (0.57, 0.82)	0.28 (0.18, 0.43)	1
Left thalamus	0	0.85 (0.77, 0.91)	0.15 (0.09, 0.23)	0.34

**Table 6:** Results of Pearson’s correlation analysis between CAT12 and volBrain pipelines for subcortical volume measurement. *p*-Values less than 0.05 were considered to be significant. Significant *p*-values are marked with an asterisk *.

	Pearson’s Correlation Coefficient (*r*)	*p*-Value
Right amygdala	0.89	< 0.001 *
Left amygdala	0.85	< 0.001 *
Right caudate	0.90	< 0.001 *
Left caudate	0.91	< 0.001 *
Right pallidum	0.94	< 0.001 *
Left pallidum	0.92	< 0.001 *
Right putamen	0.93	< 0.001 *
Left putamen	0.93	< 0.001 *
Right thalamus	0.74	< 0.001 *
Left thalamus	0.79	< 0.001 *
Right accumbens	0.85	< 0.001 *
Left accumbens	0.86	< 0.001 *

## Data Availability

Data are available upon request.

## Supplementary Materials

The following supporting information can be downloaded at: https://www.mdpi.com/article/10.3390/medicina58111687/s1, Supplement S1: Information on segmentation pipelines; Table S1: Detailed ACE analysis. References [31,32,37,53,54,55,56,57,58,59] are cited in the supplementary materials.
