# Supplementary material for: Heritability of Subcortical Grey Matter Structures
Source: Medicina (Kaunas). 2022 Nov 21;58(11):1687. doi: 10.3390/medicina58111687 (PMC9696305; doi:10.3390/medicina58111687)
Supplement: Supplementary file 1 [file medicina-58-01687-s001.zip › SupplementS1.pdf]

Currently, manual segmentation techniques are considered the gold standard for volumetric quantification of regional brain structures. However, these methods present substantial limitations, which make their use for larger data sets less feasible. These limitations include the heavy time burden that is required for manual segmentation and the need for trained personnel with expertise in the methodology. These tasks are also operator and rate-dependent and, without strict protocols, can lead to variable results. Due to the limitations of manual segmentation, automated approaches for brain processing and segmentation have been increasingly implemented. They allow for volumetric quantification of large MRI datasets from worldwide sources. They have the advantages of excellent reproducibility and decreased time, training, and anatomical knowledge requirements. This has enabled researchers to perform large-scale studies of brain anatomy and related disease progressions [1].

Some of the better known and more commonly used software include FreeSurfer [2] (Martinos Center for Biomedical Imaging, Harvard-MIT, Boston; available at http://surfer.nmr.mgh.harvard.edu/), "FIRST" which is part of the FMRIB Software Library (FSL) [3] (Analysis Group, FMRIB, Oxford, United Kingdom; available at http://fsl.fmrib. ox.ac.uk/fsl/fslwiki/), and Statistical Parametric Mapping 12 (SPM12) [4] (Wellcome Department of Cognitive Neurology, Institute of Neurology, Queen Square, London; available at http://www.fil.ion.ucl.ac.uk/spm/). Other more recent software include volBrain [5] (http://volbrain.upv.es, Manjón and Coupé 2016) and the Computational Anatomy Toolbox 12 (CAT12) [6] (Christian Gaser 2018, http://www.neuro.uni-jena.de/cat/) which is an extension of the previously mentioned SPM12.

FreeSurfer functions by automatically assigning a label to each voxel from anatomical images based on probabilistic estimations which rely on Markov random fields. It can be considered a semi-automated method, as it allows users to edit the quality of the automatically performed segmentation. At the same time, FSL uses a probabilistic framework with boundaries of brain structures based on signal intensity of T1-weighted images and the expected shape of structures to be segmented. SPM12 uses the unified segmentation algorithm, which combines tissue classification, bias correction, and image registration in the same generative model. It bases the image segmentation on Tissue Probability Maps (TPM), which represent the prior probability of an image unit (voxel) being either GM, WM, or non-brain tissue. In contrast, CAT12 uses TPM only to spatially normalize the image, perform an initial skull-stripping, and initialize the segmentation. Then, it uses an adaptive maximum a posteriori segmentation approach. Lastly, volBrain employs a fully automated multi-atlas label fusion approach to segmentation and volume estimation [1, 7, 8].

When comparing the different automated methods and manual segmentation, results vary based on the structures being examined. A study by Akudjedu et al. showed a high correlation between automated methods (FreeSurfer, FSL, volBrain) and manual segmentation for easily segmented structures such as the caudate nucleus and a moderate correlation for complex structures such as the hippocampus. Additionally, absolute volumes were found to be relatively accurate for easy-to-segment structures, but difficult-to-segment structures showed a poor approximation of absolute volumes. All automated methods overestimated the volume of the hippocampus but underestimated the volume of the caudate nucleus [1]. Another study examined the robustness of brain volume measurements across varying magnetic field strengths for FSL, SPM, and FreeSurfer. Results showed that robustness varied for different structures measured. TBV, GM, and WM volumes were robust for FreeSurfer and FSL but less robust for SPM. However, ICV was more robust for SPM than FSL and FreeSurfer. Additionally, this study examined the accuracy of volume measurements in comparison to manual segmentation. Findings showed that for GM volume, SPM was the most accurate method, for WM volume, FreeSurfer was the most accurate method,

and for ICV, FSL was the most accurate method [7]. A comparison between the CAT12 pipeline and SPM12, performed by Tavares et al., demonstrated that the volume estimates were highly correlated for the two methods. However, absolute volume differences were seen to be tissue specific. CAT12 appeared to overestimate total GM relative volumes and underestimate total WM relative volumes with increasing mean volumes in healthy adults. In addition, the study compared the ability of both pipelines to distinguish patients with Alzheimer's disease (AD) from healthy adults. Results obtained demonstrated that both methods were able to produce volumes detecting AD above chance level. This was observed most profoundly in the relative hippocampal volumes. Furthermore, as demonstrated in previous studies, the ability to detect AD did not differ significantly between methods [8, 9].

The automated volumetric tools mentioned so far have mainly been used in the realm of scientific research. However, in recent years the U.S. Food and Drug Administration (FDA) has approved several commercially available automated volumetric software for use in clinical settings. The most widely used is NeuroQuant® (CorTechs Labs) [10]. It has been approved for use in detecting hippocampal and medial temporal lobe atrophy in AD. Furthermore, it has been reported to be useful in evaluating patients with progression of mild cognitive impairment, traumatic brain injury, and epilepsy [11, 12].

References:

1. Akudjedu, T.N.; Nabulsi, L.; Makelyte, M.; Scanlon, C.; Hehir, S.; Casey, H.; Ambati, S.; Kenney, J.; O'Donoghue, S.; McDermott, E.; et al. A comparative study of segmentation techniques for the quantification of brain subcortical volume. *Brain Imaging Behav.* **2018**, *12*, 1678–1695.
2. Fischl, B.; Salat, D.H.; Busa, E.; Albert, M.; Dieterich, M.; Haselgrove, C.; van der Kouwe, A.; Killiany, R.; Kennedy, D.; Klaveness, S.; et al. Whole brain segmentation: Automated labeling of neuroanatomical structures in the human brain. *Neuron* **2002**, *33*, 341–355.
3. Smith, S.M.; Zhanga, Y.; Jenkinson, M.; Chenab, J.; Matthews, P.M.; Federico, A.; de Stefano, N. Accurate, robust, and automated longitudinal and cross-sectional brain change analysis. *Neuroimage* **2002**, *17*, 479–489.
4. Ashburner, J.; Friston, K.J. Unified segmentation. Neuroimage 2005, 26, 839–851.
5. Manjon, J.V.; Coupe, P. volBrain: An Online MRI Brain Volumetry System. Front. Neuroinform. 2016, 10, 30.
6. Gaser, C.; Dahnke, R.; Thompson, P.M.; Kurth, F.; Luders, E.; Alzheimer's Disease Neuroimaging Initiative. A Computational Anatomy Toolbox for the Analysis of Structural MRI Data. Neuroimage, in review. BioRxiv 2022. https://doi.org/10.1101/2022.06.11.495736
7. Heinen, R.; Bouvy, W.H.; Mendrik, A.M.; Viergever, M.A.; Biessels, G.J.; de Bresser, J. Robustness of Automated Methods for Brain Volume Measurements across Different MRI Field Strengths. *PLoS ONE* **2016**, *11*, e0165719.
8. Tavares, V.; Prata, D.; Ferreira, H.A. Comparing SPM12 and CAT12 segmentation pipelines: A brain tissue volume-based age and Alzheimer's disease study. *J. Neurosci. Methods* **2019**, *334*, 108565.
9. Buchert, R.; Lange, C.; Suppa, P.; Apostolova, I.; Spies, L.; Teipel, S.; Dubois, B.; Hampel, H.; Grothe, M.J. Magnetic resonance imaging-based hippocampus volume for prediction of dementia in mild cognitive impairment: Why does the measurement method matter so little? *Alzheimers Dement.* **2018**, *14*, 976–978.
10. Brewer, J.B.; Magda, S.; Airriess, C.; Smith, M. Fully-automated quantification of regional brain volumes for improved detection of focal atrophy in Alzheimer disease. *AJNR Am. J. Neuroradiol.* **2009**, *30*, 578–580.

11. Lee, J.Y.; Oh, S.W.; Chung, M.S.; Park, J.E.; Moon, Y.; Jeon, H.J.; Moon, W.-J. Clinically Available Software for Automatic Brain Volumetry: Comparisons of Volume Measurements and Validation of Intermethod Reliability. *Korean J. Radiol.* **2021**, *22*, 405–414.
12. Ochs, A.L.; Ross, D.E.; Zannoni, M.D.; Abildskov, T.J.; Bigler, E.D.; Initiative, F.T.A.D.N. Comparison of Automated Brain Volume Measures obtained with NeuroQuant and FreeSurfer. *J. Neuroimaging* **2015**, *25*, 721–727.
