# Supplementary material for: Heritability of Subcortical Grey Matter Structures
Source: Medicina (Kaunas). 2022 Nov 21;58(11):1687. doi: 10.3390/medicina58111687 (PMC9696305; doi:10.3390/medicina58111687)
Supplement: Supplementary file 1 [file medicina-58-01687-s001.zip › TableS1.pdf]

CAT12 Analysis

*AIC: akaike information criteria, BIC: bayesian information criteria, -2LL: -2*loglikelihood value, df: degree of freedom, DiffLL: the difference of the -2LL of the base model (ACE) and the reduced model in the given row (AE/CE/E), P value: p value of the likelihood ratio test, best fitting model is marked with a star (*).*

**TIV cm3**	**AIC**	**BIC**	**-2LL**	**df**	**DiffLL**	**P value**	**Results (95% CI)**
ACE	1,466.164	1,457.102	1450.128	7	Reference	Reference	A: 0.932 (0.894, 0.955)<br>C: 0 (0, 0.106)<br>E: 0.068 (0.045, 0.106)
AE*	1,463.628	1,456.106	1450.128	6	0	1	A: 0.932 (0.894, 0.955)<br>C: 0<br>E: 0.068 (0.045, 0.106)
CE	1,497.725	1,490.203	1484.225	6	-34.097	0	A: 0<br>C: 0.759 (0.657, 0.846)<br>E: 0.241 (0.154, 0.343)
E	1,549.457	1,543.387	1538.405	5	-88.277	0	A: 0<br>C: 0<br>E: 1
SAT	1,472.571	1,458.303	Rmz: 0.924 (0.881, 0.949)	Rdz: 0.501 (0.099, 0.72)			

**Total GM cm3**	**AIC**	**BIC**	**-2LL**	**df**	**DiffLL**	**P value**	**Results (95% CI)**
ACE	1,273.104	1,264.042	1257.068	7	Reference	Reference	A: 0.925 (0.882, 0.956)<br>C: 0 (0, 0.118)<br>E: 0.075 (0.044, 0.118)
AE*	1,270.568	1,263.046	1257.068	6	0	1	A: 0.925 (0.882, 0.956)<br>C: 0<br>E: 0.075 (0.044, 0.118)
CE	1,309.797	1,302.275	1296.297	6	-39.229	0	A: 0<br>C: 0.67 (0.509, 0.765)<br>E: 0.33 (0.235, 0.491)
E	1,344.891	1,338.820	1333.838	5	-76.77	0	A: 0<br>C: 0<br>E: 1
SAT	1,278.755	1,264.488	Rmz: 0.912 (0.847, 0.941)	Rdz: 0.271 (-0.164, 0.586)			

**Total WM cm3**	**AIC**	**BIC**	**-2LL**	**df**	**DiffLL**	**P value**	**Results (95% CI)**
ACE	1,275.221	1,266.159	1259.185	7	Reference	Reference	A: 0.931 (0.892, 0.958)<br>C: 0 (0, 0.108)<br>E: 0.069 (0.042, 0.108)
AE*	1,272.685	1,265.163	1259.185	6	0	1	A: 0.931 (0.892, 0.958)<br>C: 0<br>E: 0.069 (0.042, 0.108)
CE	1,305.383	1,297.861	1291.883	6	-32.698	0	A: 0<br>C: 0.78 (0.685, 0.843)<br>E: 0.22 (0.157, 0.315)
E	1,361.916	1,355.845	1350.864	5	-91.679	0	A: 0<br>C: 0<br>E: 1
SAT	1,282.982	1,268.715	Rmz: 0.934 (0.896, 0.956)	Rdz: 0.414 (0.012, 0.671)			

**Total CSF cm3**	**AIC**	**BIC**	**-2LL**	**df**	**DiffLL**	**P value**	**Results (95% CI)**
ACE	1,335.221	1,326.159	1319.185	7	Reference	Reference	A: 0.779 (0.301, 0.884)<br>C: 0.029 (0, 0.327)<br>E: 0.192 (0.114, 0.327)
AE*	1,332.696	1,325.174	1319.196	6	-0.011	0.916	A: 0.809 (0.674, 0.886)<br>C: 0<br>E: 0.191 (0.114, 0.326)

CE	1,342.949	1,335.427	1329.449	6	-10.264	0.001	A: 0<br>C: 0.614 (0.435, 0.746)<br>E: 0.386 (0.254, 0.565)
E	1,370.255	1,364.184	1359.203	5	-40.018	0	A: 0<br>C: 0<br>E: 1
SAT	1,315.208	1,300.940	Rmz: 0.665 (0.458, 0.801)	Rdz: 0.731 (0.493, 0.849)			

**Right Accumbens cm3**	**AIC**	**BIC**	**-2LL**	**df**	**DiffLL**	**P value**	**Results (95% CI)**
ACE	-524.461	-533.523	-540.497	7	Reference	Reference	A: 0.902 (0.576, 0.942)<br>C: 0 (0, 0.168)<br>E: 0.098 (0.058, 0.168)
AE*	-526.997	-534.519	-540.497	6	0	1	A: 0.902 (0.855, 0.941)<br>C: 0<br>E: 0.098 (0.059, 0.145)
CE	-498.013	-505.535	-511.513	6	-28.985	0	A: 0<br>C: 0.698 (0.547, 0.801)<br>E: 0.302 (0, 0.452)
E	-458.369	-464.440	-469.421	5	-71.076	0	A: 0<br>C: 0<br>E: 1
SAT	-517.366	-531.634	Rmz: 0.898 (0.82, 0.943)	Rdz: 0.289 (-0.15, 0.635)			

**Left Accumbens cm3**	**AIC**	**BIC**	**-2LL**	**df**	**DiffLL**	**P value**	**Results (95% CI)**
ACE	-462.532	-471.594	-478.568	7	Reference	Reference	A: 0.908 (0.775, 0.947)<br>C: 0 (0, 0.167)<br>E: 0.092 (0.053, 0.167)
AE*	-465.068	-472.590	-478.568	6	0	1	A: 0.908 (0.833, 0.947)<br>C: 0<br>E: 0.092 (0.053, 0.167)
CE	-429.242	-436.764	-442.742	6	-35.826	0	A: 0<br>C: 0.495 (0.286, 0.659)<br>E: 0.505 (0.341, 0.714)
E	-413.995	-420.066	-425.048	5	-53.52	0	A: 0<br>C: 0<br>E: 1
SAT	-470.663	-484.931	Rmz: 0.862 (0.761, 0.922)	Rdz: 0.111 (-0.332, 0.515)			

**Right Amygdala cm3**	**AIC**	**BIC**	**-2LL**	**df**	**DiffLL**	**P value**	**Results (95% CI)**
ACE	-335.001	-344.063	-351.037	7	Reference	Reference	A: 0.855 (0.353, 0.912)<br>C: 0 (0, 0.241)<br>E: 0.145 (0.088, 0.241)
AE*	-337.537	-345.059	-351.037	6	0	1	A: 0.855 (0.759, 0.912)<br>C: 0<br>E: 0.145 (0.088, 0.241)
CE	-322.601	-330.123	-336.101	6	-14.937	0	A: 0<br>C: 0.723 (0.582, 0.822)<br>E: 0.277 (0.178, 0.418)
E	-278.435	-284.505	-289.487	5	-61.55	0	A: 0<br>C: 0<br>E: 1
SAT	-330.684	-344.951	Rmz: 0.873 (0.777, 0.929)	Rdz: 0.358 (-0.079, 0.683)			

**Left Amygdala cm3**	**AIC**	**BIC**	**-2LL**	**df**	**DiffLL**	**P value**	**Results (95% CI)**
ACE	-283.247	-292.309	-299.284	7	Reference	Reference	A: 0.754 (0.436, 0.856)<br>C: 0 (0, 0.42)<br>E: 0.246 (0.144, 0.42)
AE*	-285.784	-293.306	-299.284	6	0	1	A: 0.754 (0.579, 0.856)<br>C: 0

							E: 0.246 (0.144, 0.421)
CE	-273.046	-262.391	-286.546	6	-12.738	0	A: 0<br>C: 0.512 (0.307, 0.673)<br>E: 0.488 (0.327, 0.693)
E	-256.320	-284.505	-267.373	5	-31.911	0	A: 0<br>C: 0<br>E: 1
SAT	-282.165	-296.432	Rmz: 0.737 (0.563, 0.849)	Rdz: 0.161 (-0.299, 0.563)			

**Right Caudate cm3**	**AIC**	**BIC**	**-2LL**	**df**	**DiffLL**	**P value**	**Results (95% CI)**
ACE	40.596	31.534	24.559	7	Reference	Reference	A: 0.429 (0.099, 0.905)<br>C: 0.443 (0, 0.216)<br>E: 0.128 (0.077, 0.216)
AE*	40.268	32.746	26.768	6	-2.209	0.137	A: 0.87 (0.786, 0.92)<br>C: 0<br>E: 0.13 (0.08, 0.214)
CE	45.291	37.769	31.791	6	-7.232	0.007	A: 0<br>C: 0.804 (0.697, 0.876)<br>E: 0.196 (0.124, 0.303)
E	108.278	102.207	97.226	5	-72.666	0	A: 0<br>C: 0<br>E: 1
SAT	48.189	33.922	Rmz: 0.873 (0.778, 0.929)	Rdz: 0.659 (0.323, 0.848)			

**Left Caudate cm3**	**AIC**	**BIC**	**-2LL**	**df**	**DiffLL**	**P value**	**Results (95% CI)**
ACE	29.068	20.006	13.031	7	Reference	Reference	A: 0.906 (0.482, 0.943)<br>C: 0 (0, 0.16)<br>E: 0.094 (0.057, 0.16)
AE*	26.531	19.009	13.031	6	0	1	A: 0.906 (0.84, 0.943)<br>C: 0<br>E: 0.094 (0.057, 0.16)
CE	52.686	45.164	39.186	6	-26.155	0	A: 0<br>C: 0.736 (0.599, 0.83)<br>E: 0.264 (0.17, 0.401)
E	99.353	93.282	88.3	5	-75.269	0	A: 0<br>C: 0<br>E: 1
SAT	36.355	22.087	Rmz: 0.9 (0.823, 0.944)	Rdz: 0.442 (0.016, 0.736)			

**Right Pallidum cm3**	**AIC**	**BIC**	**-2LL**	**df**	**DiffLL**	**P value**	**Results (95% CI)**
ACE	-233.753	-242.815	-249.789	7	Reference	Reference	A: 0.834 (0.351, 0.941)<br>C: 0.069 (0, 0.163)<br>E: 0.097 (0.058, 0.163)
AE*	-236.253	-243.775	-249.753	6	-0.036	0.85	A: 0.903 (0.837, 0.941)<br>C: 0<br>E: 0.097 (0.059, 0.163)
CE	-215.879	-223.401	-229.379	6	-20.41	0	A: 0<br>C: 0.787 (0.672, 0.865)<br>E: 0.213 (0.135, 0.328)
E	-157.554	-163.625	-168.607	5	-81.182	0	A: 0<br>C: 0<br>E: 1
SAT	-227.225	-241.493	Rmz: 0.913 (0.846, 0.951)	Rdz: 0.432 (0.016, 0.721)			

**Left Pallidum cm3**	**AIC**	**BIC**	**-2LL**	**df**	**DiffLL**	**P value**	**Results (95% CI)**
ACE	-198.684	-207.746	-214.72	7	Reference	Reference	A: 0.926 (0.713, 0.956)<br>C: 0 (0, 0.13)<br>E: 0.074 (0.044, 0.13)
AE*	-201.220	-208.742	-214.72	6	0	1	A: 0.926 (0.87, 0.956)<br>C: 0

							E: 0.074 (0.044, 0.13)
CE	-160.016	-167.538	-173.516	6	-41.204	0	A: 0<br>C: 0.614 (0.435, 0.746)<br>E: 0.386 (0.254, 0.565)
E	-132.680	-138.751	-143.732	5	-70.988	0	A: 0<br>C: 0<br>E: 1
SAT	-199.364	-213.632	Rmz: 0.904 (0.831, 0.946)	Rdz: 0.25 (-0.195, 0.613)			

**Right Putamen cm3**	**AIC**	**BIC**	**-2LL**	**df**	**DiffLL**	**P value**	**Results (95% CI)**
ACE	50.606	41.544	34.57	7	Reference	Reference	A: 0.898 (0.632, 0.939)<br>C: 0 (0, 0.177)<br>E: 0.102 (0.061, 0.177)
AE*	48.070	40.548	34.57	6	0	1	A: 0.898 (0.823, 0.939)<br>C: 0 (0, 1)<br>E: 0.102 (0.061, 0.177)
CE	79.053	71.531	65.553	6	-30.983	0	A: 0 (0, 1)<br>C: 0.653 (0.488, 0.774)<br>E: 0.347 (0.226, 0.512)
E	111.685	105.614	100.633	5	-66.063	0	A: 0 (0, 1)<br>C: 0 (0, 1)<br>E: 1 (0, 1)
SAT	52.667	38.400	Rmz: 0.898 (0.821, 0.943)	Rdz: 0.195 (-0.243, 0.568)			

**Left Putamen cm3**	**AIC**	**BIC**	**-2LL**	**df**	**DiffLL**	**P value**	**Results (95% CI)**
ACE	106.219	97.157	90.183	7	Reference	Reference	A: 0.951 (0.875, 0.971)<br>C: 0 (0, 0.071)<br>E: 0.049 (0.029, 0.071)
AE*	103.683	96.161	90.183	6	0	1	A: 0.951 (0.932, 0.971)<br>C: 0<br>E: 0.049 (0.029, 0.068)
CE	158.086	150.564	144.586	6	-54.404	0	A: 0<br>C: 0.42 (0.197, 0.602)<br>E: 0.58 (0.398, 0.803)
E	167.895	161.824	156.842	5	-66.66	0	A: 0<br>C: 0<br>E: 1
SAT	79.481	65.214	Rmz: 0.919 (0.857, 0.955)	Rdz: 0.017 (-0.41, 0.437)			

**Right Thalamus cm3**	**AIC**	**BIC**	**-2LL**	**df**	**DiffLL**	**P value**	**Results (95% CI)**
ACE	193.987	184.925	177.951	7	Reference	Reference	A: 0.07 (0, 0.642)<br>C: 0.673 (0.117, 0.404)<br>E: 0.257 (0.156, 0.404)
AE	196.495	188.973	182.995	6	-5.044	0.025	A: 0.751 (0.61, 0.843)<br>C: 0<br>E: 0.249 (0.157, 0.39)
CE*	191.564	184.042	178.064	6	-0.113	0.737	A: 0<br>C: 0.731 (0.593, 0.828)<br>E: 0.269 (0.172, 0.407)
E	237.340	231.269	226.287	5	-48.336	0	A: 0<br>C: 0<br>E: 1
SAT	200.726	186.458	Rmz: 0.741 (0.572, 0.85)	Rdz: 0.727 (0.434, 0.881)			

**Left Thalamus cm3**	**AIC**	**BIC**	**-2LL**	**df**	**DiffLL**	**P value**	**Results (95% CI)**
ACE*	156.545	147.483	140.508	7	Reference	Reference	A: 0.44 (0.127, 0.914)<br>C: 0.447 (0, 0.192)<br>E: 0.113 (0.067, 0.192)
AE	156.599	149.077	143.099	6	-2.591	0.108	A: 0.886 (0.811, 0.93)<br>C: 0

							E: 0.114 (0.07, 0.189)
CE	162.840	155.318	149.34	6	-8.832	0.003	A: 0<br>C: 0.812 (0.71, 0.882)<br>E: 0.188 (0.118, 0.29)
E	227.829	221.758	216.776	5	-76.268	0	A: 0<br>C: 0<br>E: 1
SAT	163.022	148.755	Rmz: 0.873<br>(0.778, 0.929)	Rdz: 0.724<br>(0.422,0.881)			

volBrain Analysis

*AIC: akaike information criteria, BIC: bayesian information criteria, -2LL: -2*loglikelihood value, df: degree of freedom, DiffLL: the difference of the -2LL of the base model (ACE) and the reduced model in the given row (AE/CE/E), P value: p value of the likelihood ratio test, best fitting model is marked with a star (*).*

**TIV cm3**	**AIC**	**BIC**	**-2LL**	**df**	**DiffLL**	**P value**	**Results (95% CI)**
ACE	1,462.343	1,453.281	1446.306	7	Reference	Reference	A: 0.934 (0.896, 0.956)<br>C: 0 (0, 0.104)<br>E: 0.066 (0.044, 0.104)
AE*	1,459.806	1,452.284	1446.306	6	0	1	A: 0.934 (0.896, 0.956)<br>C: 0<br>E: 0.066 (0.044, 0.104)
CE	1,499.379	1,491.857	1485.879	6	-39.572	0	A: 0<br>C: 0.728 (0.613, 0.825)<br>E: 0.272 (0.175, 0.387)
E	1,544.482	1,538.412	1533.43	5	-87.124	0	A: 0<br>C: 0<br>E: 1
SAT	1,469.257	1,454.990	Rmz: 0.929<br>(0.888, 0.952)	Rdz: 0.356<br>(-0.06, 0.636)			

**Total GM cm3**	**AIC**	**BIC**	**-2LL**	**df**	**DiffLL**	**P value**	**Results (95% CI)**
ACE	1,261.700	1,252.638	1245.664	7	Reference	Reference	A: 0.937 (0.9, 0.962)<br>C: 0<br>E: 0.063 (0.038, 0.1)
AE*	1,259.164	1,251.642	1245.664	6	0	1	A: 0.937 (0.9, 0.962)<br>C: 0<br>E: 0.063 (0.038, 0.1)
CE	1,301.161	1,293.639	1287.661	6	-41.998	0	A: 0<br>C: 0.727 (0.611, 0.824)<br>E: 0.273 (0.176, 0.389)
E	1,346.079	1,340.008	1335.027	5	-89.363	0	A: 0<br>C: 0<br>E: 1
SAT	1,268.324	1,254.057	Rmz: 0.936<br>(0.886, 0.957)	Rdz: 0.268<br>(-0.159, 0.584)			

**Total WM cm3**	**AIC**	**BIC**	**-2LL**	**df**	**DiffLL**	**P value**	**Results (95% CI)**
ACE	1,337.396	1,328.334	1321.36	7	Reference	Reference	A: 0.881 (0.799, 0.921)<br>C: 0 (0, 0.201)<br>E: 0.119 (0.079, 0.201)
AE*	1,334.860	1,327.338	1321.36	6	0	1	A: 0.881 (0.799, 0.921)<br>C: 0<br>E: 0.119 (0.079, 0.201)
CE	1,355.013	1,347.491	1341.513	6	-20.153	0	A: 0<br>C: 0.719 (0.576, 0.799)<br>E: 0.281 (0.201, 0.424)
E	1,398.390	1,392.319	1387.337	5	-65.978	0	A: 0<br>C: 0<br>E: 1

SAT	1,345.189	1,330.922	Rmz: 0.875 (0.805, 0.93)	Rdz: 0.418 (0.018, 0.673)			

Total CSF cm3	AIC	BIC	-2LL	df	DiffLL	P value	Results (95% CI)
ACE	-44.793	-53.855	-60.829	7	Reference	Reference	A: 0.427 (0, 0.834)<br>C: 0.317 (0, 0.409)<br>E: 0.256 (0.158, 0.409)
AE*	-46.745	-54.267	-60.245	6	-0.584	0.445	A: 0.744 (0.598, 0.839)<br>C: 0<br>E: 0.256 (0.161, 0.402)
CE	-44.954	-52.476	-58.454	6	-2.375	0.123	A: 0<br>C: 0.692 (0.538, 0.802)<br>E: 0.308 (0.198, 0.462)
E	-7.017	-13.088	-18.07	5	-42.759	0	A: 0<br>C: 0<br>E: 1
SAT	-40.265	-54.532	Rmz: 0.79 (0.638, 0.883)	Rdz: 0.344 (-0.132, 0.705)			

Right Accumbens cm3	AIC	BIC	-2LL	df	DiffLL	P value	Results (95% CI)
ACE	-404.850	-414.058	-420.924	7	Reference	Reference	A: 0.694 (0.375, 0.82)<br>C: 0 (0, 0.515)<br>E: 0.306 (0.18, 0.515)
AE*	-407.396	-415.039	-420.924	6	0	1	A: 0.694 (0.485, 0.82)<br>C: 0<br>E: 0.306 (0.18, 0.515)
CE	-396.784	-404.427	-410.312	6	-10.612	0.001	A: 0<br>C: 0.453 (0.234, 0.628)<br>E: 0.547 (0.372, 0.766)
E	-385.014	-391.181	-396.085	5	-24.839	0	A: 0<br>C: 0<br>E: 1
SAT	-407.325	-421.830	Rmz: 0.724 (0.545, 0.84)	Rdz: -0.015 (-0.446, 0.424)			

Left Accumbens cm3	AIC	BIC	-2LL	df	DiffLL	P value	Results (95% CI)
ACE	-426.733	-435.941	-442.807	7	Reference	Reference	A: 0.775 (0.49, 0.867)<br>C: 0 (0, 0.383)<br>E: 0.225 (0.133, 0.383)
AE*	-429.280	-436.922	-442.807	6	0	1	A: 0.775 (0.617, 0.867)<br>C: 0<br>E: 0.225 (0.133, 0.383)
CE	-413.905	-421.547	-427.432	6	-15.375	0	A: 0<br>C: 0.541 (0.341, 0.694)<br>E: 0.459 (0.306, 0.659)
E	-394.859	-401.027	-405.931	5	-36.876	0	A: 0<br>C: 0<br>E: 1
SAT	-422.723	-437.229	Rmz: 0.787 (0.64, 0.879)	Rdz: -0.068 (-0.49, 0.384)			

Right Amygdala cm3	AIC	BIC	-2LL	df	DiffLL	P value	Results (95% CI)
ACE	-269.808	-279.016	-285.882	7	Reference	Reference	A: 0.375 (0, 0.863)<br>C: 0.427 (0, 0.325)<br>E: 0.198 (0.12, 0.325)
AE*	-270.783	-278.426	-284.311	6	-1.571	0.21	A: 0.802 (0.682, 0.877)<br>C: 0<br>E: 0.198 (0.123, 0.318)
CE	-269.277	-276.920	-282.805	6	-3.077	0.079	A: 0

							C: 0.749 (0.617, 0.84)<br>E: 0.251 (0.16, 0.383)
E	-220.753	-226.920	-231.824	5	-54.057	0	A: 0<br>C: 0<br>E: 1
SAT	-266.211	-280.717	Rmz: 0.825 (0.7, 0.901)	Rdz: 0.591 (0.207, 0.818)			

**Left Amygdala cm3**	**AIC**	**BIC**	**-2LL**	**df**	**DiffLL**	**P value**	**Results (95% CI)**
ACE	-268.730	-277.938	-284.804	7	Reference	Reference	A: 0.129 (0, 0.729)<br>C: 0.647 (0.064, 0.363)<br>E: 0.224 (0.136, 0.363)
AE	-266.948	-274.590	-280.475	6	-4.329	0.037	A: 0.777 (0.649, 0.86)<br>C: 0<br>E: 0.223 (0.14, 0.351)
CE*	-270.844	-278.486	-284.371	6	-0.433	0.51	A: 0<br>C: 0.756 (0.628, 0.845)<br>E: 0.244 (0.155, 0.372)
E	-220.643	-226.810	-231.715	5	-53.09	0	A: 0<br>C: 0<br>E: 1
SAT	-262.435	-276.941	Rmz: 0.783 (0.635, 0.876)	Rdz: 0.712 (0.397, 0.879)			

**Right Caudate cm3**	**AIC**	**BIC**	**-2LL**	**df**	**DiffLL**	**P value**	**Results (95% CI)**
ACE	85.548	76.340	69.474	7	Reference	Reference	A: 0.553 (0.179, 0.92)<br>C: 0.328 (0, 0.202)<br>E: 0.12 (0.071, 0.202)
AE*	84.022	76.379	70.494	6	-1.02	0.313	A: 0.878 (0.798, 0.926)<br>C: 0<br>E: 0.122 (0.074, 0.202)
CE	93.453	85.810	79.925	6	-10.451	0.001	A: 0<br>C: 0.798 (0.688, 0.873)<br>E: 0.202 (0.127, 0.312)
E	153.924	147.757	142.853	5	-73.378	0	A: 0<br>C: 0<br>E: 1
SAT	92.944	78.439	Rmz: 0.886 (0.799, 0.936)	Rdz: 0.588 (0.207, 0.816)			

**Left Caudate cm3**	**AIC**	**BIC**	**-2LL**	**df**	**DiffLL**	**P value**	**Results (95% CI)**
ACE	65.614	56.406	49.54	7	Reference	Reference	A: 0.464 (0.156, 0.933)<br>C: 0.446 (0, 0.154)<br>E: 0.09 (0.054, 0.154)
AE*	65.296	57.654	51.769	6	-2.228	0.135	A: 0.907 (0.845, 0.943)<br>C: 0<br>E: 0.093 (0.057, 0.155)
CE	74.578	66.936	61.051	6	-11.511	0.001	A: 0<br>C: 0.84 (0.749, 0.9)<br>E: 0.16 (0.1, 0.251)
E	148.055	141.887	136.983	5	-87.443	0	A: 0<br>C: 0<br>E: 1
SAT	72.947	58.442	Rmz: 0.912 (0.843, 0.951)	Rdz: 0.677 (0.343, 0.86)			

**Right Pallidum cm3**	**AIC**	**BIC**	**-2LL**	**df**	**DiffLL**	**P value**	**Results (95% CI)**
ACE	-166.406	-175.614	-182.48	7	Reference	Reference	A: 0.515 (0.102, 0.899)<br>C: 0.332 (0, 0.253)<br>E: 0.153 (0.093, 0.253)
AE*	-168.154	-175.796	-181.681	6	-0.799	0.371	A: 0.844 (0.747, 0.904)<br>C: 0<br>E: 0.156 (0.096, 0.253)
CE	-162.315	-169.957	-175.842	6	-6.638	0.01	A: 0

							C: 0.781 (0.663, 0.861)<br>E: 0.219 (0.139, 0.337)
E	-106.355	-112.523	-117.427	5	-65.053	0	A: 0<br>C: 0<br>E: 1
SAT	-160.074	-174.580	Rmz: 0.865<br>(0.766, 0.924)	Rdz: 0.491<br>(0.079, 0.761)			

**Left Pallidum cm3**	**AIC**	**BIC**	**-2LL**	**df**	**DiffLL**	**P value**	**Results (95% CI)**
ACE	-160.090	-169.299	-176.164	7	Reference	Reference	A: 0.682 (0.21, 0.904)<br>C: 0.163 (0, 0.258)<br>E: 0.155 (0.094, 0.258)
AE*	-162.423	-170.066	-175.951	6	-0.214	0.644	A: 0.844 (0.745, 0.905)<br>C: 0<br>E: 0.156 (0.095, 0.255)
CE	-152.695	-160.338	-166.223	6	-9.942	0.002	A: 0<br>C: 0.746 (0.612, 0.838)<br>E: 0.254 (0.162, 0.388)
E	-104.819	-110.986	-115.89	5	-60.274	0	A: 0<br>C: 0<br>E: 1
SAT	-152.841	-167.347	Rmz: 0.85<br>(0.742, 0.915)	Rdz: 0.496<br>(0.083, 0.763)			

**Right Putamen cm3**	**AIC**	**BIC**	**-2LL**	**df**	**DiffLL**	**P value**	**Results (95% CI)**
ACE	70.186	60.977	54.112	7	Reference	Reference	A: 0.922 (0.674, 0.954)<br>C: 0 (0, 0.136)<br>E: 0.078 (0.046, 0.136)
AE*	67.639	59.996	54.112	6	0	1	A: 0.922 (0.864, 0.954)<br>C: 0<br>E: 0.078 (0.046, 0.136)
CE	106.391	98.749	92.864	6	-38.752	0	A: 0<br>C: 0.668 (0.504, 0.785)<br>E: 0.332 (0.215, 0.496)
E	140.554	134.387	129.483	5	-75.371	0	A: 0<br>C: 0<br>E: 1
SAT	74.806	60.301	Rmz: 0.915<br>(0.85, 0.953)	Rdz: 0.171<br>(-0.282, 0.563)			

**Left Putamen cm3**	**AIC**	**BIC**	**-2LL**	**df**	**DiffLL**	**P value**	**Results (95% CI)**
ACE	96.420	87.211	80.346	7	Reference	Reference	A: 0.897 (0.629, 0.939)<br>C: 0 (0, 0.179)<br>E: 0.103 (0.061, 0.179)
AE*	93.873	86.231	80.346	6	0	1	A: 0.897 (0.821, 0.939)<br>C: 0<br>E: 0.103 (0.061, 0.179)
CE	124.479	116.836	110.951	6	-30.606	0	A: 0<br>C: 0.653 (0.485, 0.775)<br>E: 0.347 (0.225, 0.515)
E	156.491	150.324	145.42	5	-65.074	0	A: 0<br>C: 0<br>E: 1
SAT	98.942	84.436	Rmz: 0.894<br>(0.814, 0.941)	Rdz: 0.195<br>(-0.258, 0.577)			

**Right Thalamus cm3**	**AIC**	**BIC**	**-2LL**	**df**	**DiffLL**	**P value**	**Results (95% CI)**
ACE	170.963	161.755	154.889	7	Reference	Reference	A: 0 (0, 0.272)<br>C: 0.716 (0.419, 0.429)<br>E: 0.284 (0.182, 0.429)
AE	177.798	170.156	164.271	6	-9.382	0.002	A: 0.685 (0.523, 0.796)<br>C: 0<br>E: 0.315 (0.204, 0.477)
CE*	168.416	160.774	154.889	6	0	1	A: 0

							C: 0.716 (0.57, 0.817)<br>E: 0.284 (0.183, 0.43)
E	210.456	204.289	199.385	5	-44.496	0	A: 0<br>C: 0<br>E: 1
SAT	174.572	160.066	Rmz: 0.678 (0.479, 0.81)	Rdz: 0.825 (0.61, 0.927)			

**Left Thalamus cm3**	**AIC**	**BIC**	**-2LL**	**df**	**DiffLL**	**P value**	**Results (95% CI)**
ACE	125.154	115.946	109.08	7	Reference	Reference	A: 0.11 (0, 0.485)<br>C: 0.762 (0.484, 0.218)<br>E: 0.128 (0.076, 0.218)
AE	132.179	124.537	118.652	6	-9.572	0.002	A: 0.87 (0.789, 0.919)<br>C: 0<br>E: 0.13 (0.081, 0.211)
CE*	123.507	115.865	109.98	6	-0.9	0.343	A: 0<br>C: 0.854 (0.77, 0.909)<br>E: 0.146 (0.091, 0.23)
E	202.015	195.848	190.943	5	-81.864	0	A: 0<br>C: 0<br>E: 1
SAT	129.335	114.830	Rmz: 0.862 (0.76, 0.922)	Rdz: 0.862 (0.685, 0.943)			
